# Survival-Related Profile, Pathways, and Transcription Factors in Ovarian Cancer

**DOI:** 10.1371/journal.pmed.1000024

**Published:** 2009-02-03

**Authors:** Anne P. G Crijns, Rudolf S. N Fehrmann, Steven de Jong, Frans Gerbens, Gert Jan Meersma, Harry G Klip, Harry Hollema, Robert M. W Hofstra, Gerard J. te Meerman, Elisabeth G. E de Vries, Ate G. J van der Zee

**Affiliations:** 1 Department of Gynecologic Oncology, University Medical Centre Groningen, University of Groningen, Groningen, The Netherlands; 2 Department of Medical Oncology, University Medical Centre Groningen, University of Groningen, Groningen, The Netherlands; 3 Department of Genetics, University Medical Centre Groningen, University of Groningen, Groningen, The Netherlands; 4 Department of Pathology, University Medical Centre Groningen, University of Groningen, Groningen, The Netherlands; Centre for Research in Women's Health, Canada

## Abstract

**Background:**

Ovarian cancer has a poor prognosis due to advanced stage at presentation and either intrinsic or acquired resistance to classic cytotoxic drugs such as platinum and taxoids. Recent large clinical trials with different combinations and sequences of classic cytotoxic drugs indicate that further significant improvement in prognosis by this type of drugs is not to be expected. Currently a large number of drugs, targeting dysregulated molecular pathways in cancer cells have been developed and are introduced in the clinic. A major challenge is to identify those patients who will benefit from drugs targeting these specific dysregulated pathways.The aims of our study were (1) to develop a gene expression profile associated with overall survival in advanced stage serous ovarian cancer, (2) to assess the association of pathways and transcription factors with overall survival, and (3) to validate our identified profile and pathways/transcription factors in an independent set of ovarian cancers.

**Methods and Findings:**

According to a randomized design, profiling of 157 advanced stage serous ovarian cancers was performed in duplicate using ∼35,000 70-mer oligonucleotide microarrays. A continuous predictor of overall survival was built taking into account well-known issues in microarray analysis, such as multiple testing and overfitting. A functional class scoring analysis was utilized to assess pathways/transcription factors for their association with overall survival. The prognostic value of genes that constitute our overall survival profile was validated on a fully independent, publicly available dataset of 118 well-defined primary serous ovarian cancers. Furthermore, functional class scoring analysis was also performed on this independent dataset to assess the similarities with results from our own dataset. An 86-gene overall survival profile discriminated between patients with unfavorable and favorable prognosis (median survival, 19 versus 41 mo, respectively; permutation *p*-value of log-rank statistic = 0.015) and maintained its independent prognostic value in multivariate analysis. Genes that composed the overall survival profile were also able to discriminate between the two risk groups in the independent dataset. In our dataset 17/167 pathways and 13/111 transcription factors were associated with overall survival, of which 16 and 12, respectively, were confirmed in the independent dataset.

**Conclusions:**

Our study provides new clues to genes, pathways, and transcription factors that contribute to the clinical outcome of serous ovarian cancer and might be exploited in designing new treatment strategies.

## Abstract 2

Ate van der Zee and colleagues analyze the gene expression profiles of ovarian cancer samples from 157 patients, and identify an 86-gene expression profile that seems to predict overall survival.

## Editors' Summary

**Background.:**

Ovarian cancer kills more than 100,000 women every year and is one of the most frequent causes of cancer death in women in Western countries. Most ovarian cancers develop when an epithelial cell in one of the ovaries (two small organs in the pelvis that produce eggs) acquires genetic changes that allow it to grow uncontrollably and to spread around the body (metastasize). In its early stages, ovarian cancer is confined to the ovaries and can often be treated successfully by surgery alone. Unfortunately, early ovarian cancer rarely has symptoms so a third of women with ovarian cancer have advanced disease when they first visit their doctor with symptoms that include vague abdominal pains and mild digestive disturbances. That is, cancer cells have spread into their abdominal cavity and metastasized to other parts of the body (so-called stage III and IV disease). The outlook for women diagnosed with stage III and IV disease, which are treated with a combination of surgery and chemotherapy, is very poor. Only 30% of women with stage III, and 5% with stage IV, are still alive five years after their cancer is diagnosed.

**Why Was This Study Done?:**

If the cellular pathways that determine the biological behavior of ovarian cancer could be identified, it might be possible to develop more effective treatments for women with stage III and IV disease. One way to identify these pathways is to use gene expression profiling (a technique that catalogs all the genes expressed by a cell) to compare gene expression patterns in the ovarian cancers of women who survive for different lengths of time. Genes with different expression levels in tumors with different outcomes could be targets for new treatments. For example, it might be worth developing inhibitors of proteins whose expression is greatest in tumors with short survival times. In this study, the researchers develop an expression profile that is associated with overall survival in advanced-stage serous ovarian cancer (more than half of ovarian cancers originate in serous cells, epithelial cells that secrete a watery fluid). The researchers also assess the association of various cellular pathways and transcription factors (proteins that control the expression of other proteins) with survival in this type of ovarian carcinoma.

**What Did the Researchers Do and Find?:**

The researchers analyzed the gene expression profiles of tumor samples taken from 157 patients with advanced stage serous ovarian cancer and used the “supervised principal components” method to build a predictor of overall survival from these profiles and patient survival times. This 86-gene predictor discriminated between patients with favorable and unfavorable outcomes (average survival times of 41 and 19 months, respectively). It also discriminated between groups of patients with these two outcomes in an independent dataset collected from 118 additional serous ovarian cancers. Next, the researchers used “functional class scoring” analysis to assess the association between pathway and transcription factor expression in the tumor samples and overall survival. Seventeen of 167 KEGG pathways (“wiring” diagrams of molecular interactions, reactions and relations involved in cellular processes and human diseases listed in the Kyoto Encyclopedia of Genes and Genomes) were associated with survival, 16 of which were confirmed in the independent dataset. Finally, 13 of 111 analyzed transcription factors were associated with overall survival in the tumor samples, 12 of which were confirmed in the independent dataset.

**What Do These Findings Mean?:**

These findings identify an 86-gene overall survival gene expression profile that seems to predict overall survival for women with advanced serous ovarian cancer. However, before this profile can be used clinically, further validation of the profile and more robust methods for determining gene expression profiles are needed. Importantly, these findings also provide new clues about the genes, pathways and transcription factors that contribute to the clinical outcome of serous ovarian cancer, clues that can now be exploited in the search for new treatment strategies. Finally, these findings suggest that it might eventually be possible to tailor therapies to the needs of individual patients by analyzing which pathways are activated in their tumors and thus improve survival times for women with advanced ovarian cancer.

**Additional Information.:**

Please access these Web sites via the online version of this summary at http://dx.doi.org/10.1371/journal.pmed.1000024.

- This study is further discussed in a *PLoS Medicine* Perspective by Simon Gayther and Kate Lawrenson
- See also a related *PLoS Medicine* Research Article by Huntsman and colleagues
- The US National Cancer Institute provides a brief description of what cancer is and how it develops, and information on all aspects of ovarian cancer for patients and professionals (in English and Spanish)
- The UK charity Cancerbackup provides general information about cancer, and more specific information about ovarian cancer
- MedlinePlus also provides links to other information about ovarian cancer (in English and Spanish)
- The KEGG Pathway database  provides pathway maps of known molecular networks involved in a wide range of cellular processes

## Introduction

Ovarian carcinoma is the leading cause of death from gynecologic malignancies in the Western world [1]. Debulking surgery followed by platinum-based chemotherapy is considered standard of care for patients with advanced stage ovarian cancer, but despite an initial response rate of 65%–80% to first-line chemotherapy, most patients will relapse with drug-resistant disease [2]. Consequently, the 5-y survival rate of patients with advanced-stage disease is only about 5%–30% [3].

To date, a variety of studies have employed gene expression profiling to classify ovarian carcinomas in clinically relevant subtypes [4–9]. These studies provided valuable first clues to molecular changes in serous ovarian cancer that might be exploited in new treatment strategies. However, most studies were of relatively limited size and the number of overlapping genes in the identified profiles was minimal. Although identification of gene expression profiles associated with clinically relevant subtypes in ovarian cancer is important, knowledge is now rapidly emerging on how genes interact in pathways, networks and complexes; this new information allows us to unravel the cellular pathways determining the biological behavior of ovarian cancer, and these pathways might be successfully targeted with drugs.

The aim of our study was to (1) develop a gene expression profile associated with overall survival in advanced-stage serous ovarian cancer, (2) assess the association of pathways and transcription factors with overall survival, and (3) validate our profile and identified pathways/transcription factors in a fully independent, publicly available dataset of serous ovarian cancers.

## Materials and Methods

### Patients and Tumor Samples

The study population consisted of 157 consecutive patients with advanced-stage serous ovarian cancer operated on by a gynecologic oncologist from the University Medical Center Groningen (UMCG, Groningen, The Netherlands) in the period 1990–2003. All patients were treated according to Dutch guidelines, which are based on the International Federation of Gynecology and Obstetrics (FIGO) guidelines [10,11]. Standard treatment included cytoreductive surgery followed by platinum-based chemotherapy (in combination with paclitaxel after 1996). For follow-up, patients were seen every 3 mo for the first 2 y. Thereafter, follow-up visits had an interval of 4 mo in the third year, 6 mo in the fourth and fifth year, and once a year in the sixth to tenth year. A follow-up visit comprised a general physical and gynecologic examination. CA125 serum levels were also routinely determined.

Overall survival was calculated from the date of primary surgery to the date of last follow-up (right-censored) or to the date of death due to ovarian cancer. Patients who died from intercurrent disease were right-censored at the time of death. All tumor samples were obtained at primary surgery prior to chemotherapy, flash frozen in liquid nitrogen, and stored at −80 °C. Samples were confirmed to comprise tumor cells (median percentage tumor cells: 70%, interquartile range: 50%–80%), as examined after hematoxylin and eosin staining of frozen sections. Patients gave informed consent for collection and storage of tissue samples in a tissue bank for future research. All relevant patient data were retrieved and transferred into an anonymous, password-protected, database. The patients' identity was protected by study-specific, unique patient codes and their true identity was only known to two dedicated data managers. According to Dutch regulations, these precautions meant no further institutional review board approval was needed (http://www.federa.org/).

### RNA Extraction and Amplification

Total RNA from tumor samples was subjected to cesium chloride density gradient ultracentrifugation (Roche, Almere, The Netherlands). After total RNA samples had been given DNAse treatment (Megascript T7 kit, Ambion, Huntingdon, UK), they were checked for residual DNA using a dinucleotide primer set (D11S875) specific for genomic DNA [12]. mRNA was linearly amplified by in vitro transcription using T7 RNA polymerase (Megascript T7 kit) [13]. Quality/integrity of total and amplified mRNA (cRNA) was checked by spectrophotometric analysis (criterion: UV 260/280 ratio > 1.8 for each sample), and/or agarose gel electrophoresis.

### Microarray Experiments

Two randomly selected cRNA samples were hybridized together on the arrays for intensity-based instead of ratio-based analysis of the microarray data [14]. All cRNA samples (1.5 μg) were labeled with ULS-Cy5 and ULS-Cy3 label (BIOKÉ, Leiden, The Netherlands) and hybridized to ∼35,000 70-mer two-color oligonucleotide microarrays (∼35,000 Operon v3.0 probes), manufactured by The Netherlands Cancer Institute (Amsterdam, The Netherlands, http://microarrays.nki.nl) [13]. Because each tumor sample was profiled once with Cy5 and once with Cy3, there was one replicate of the whole experiment. Samples were hybridized according to a randomized design (Figure S1) to prevent systematic biases such as those caused by batch effects or technical variation that can be introduced during labeling, hybridization, and scanning [15–17]. After randomization of the processing order of the arrays from different batches, Cy5- and Cy3-labeled cRNA samples were randomly placed onto the arrays. Arrays were scanned and expression values were calculated. The MIAME-compliant microarray data are available at http://www.ncbi.nlm.nih.gov/geo/ under accession number GSE13876.

### Quantitative Real Time-PCR

Total RNA from 31 specimens, previously extracted, isolated, and used for the microarray study, was reverse transcribed into cDNA using MMLV reverse transcriptase and hexameric random primer pd(N)6 (Invitrogen, Breda, The Netherlands). The profile of the reverse transcription reaction was 10 min at 25 °C, 50 min at 37 °C, and 15 min at 70 °C.

Quantitative real-time PCR (qRT-PCR) was performed on 12 ng of cDNA. Applied Biosystems Taqman Gene expression assays, and Taqman Universal PCR master mix (Applied Biosystems, Nieuwerkerk a/d IJssel, The Netherlands) were used to perform qRT-PCR on *FGFBP1* (Hs00183226_m1, Applied Biosystems), *TMEM45A* (Hs01046616_m1, Applied Biosystems), *FKBP7* (Hs00383941_m1, Applied Biosystems), *CCL28* (Hs00955110_m1, Applied Biosystems), and the housekeeping gene *GAPDH* (Hs02758991_g1, Applied Biosystems), which is among the most constantly expressed mRNAs [18]. All reactions were performed in 384-well plates in triplicate using an ABI PRISM 7900 HT Sequence Detection System. PCR reaction conditions were as follows: Step 1: 50 °C for 2 min, step 2: 95 °C for 10 min, step 3: 50 cycles of 95 °C for 15 s, followed by 60 °C for 1 min. We used the comparative threshold cycle (Ct) method to calculate the expression of the gene of interest relative to *GAPDH* in each sample by subtracting the mean Ct value of *GAPDH* from the mean Ct value of each gene, obtaining the ΔCt value.

### Statistical Methods

Quantile normalization was applied to log_2_-transformed Cy5 and Cy3 intensities [19]. The goal of the quantile normalization is to equalize the distribution of expression values for each array in a set of arrays: (1) expression values of each microarray were sorted, (2) median intensity in each rank across the microarrays was computed, and (3) each expression value was replaced by the median intensity at its rank.

Principal components analysis (PCA) was performed for quality control. It has been shown that the most significant principal component for a gene expression data matrix is frequently a constant pattern, which dominates the data [20]. So, the first principal component explaining the largest part of the variation could be considered variation that the arrays have in common [21,22]. Next, correlation with the first principal component was calculated for each individual array (factor loading). Factor loadings of the first principal component for an individual array can be seen as a quality index, as arrays of lesser quality would have lower or distinctly different correlations than arrays of good quality. Samples with a factor loading with the first principal components of less than 2 times the standard deviation from the mean were excluded as their hybridizations were considered to be of low quality [21,22]. Operon V3.0 probe identifiers (∼35,000) were converted to official gene symbols using probe annotations provided by The Netherlands Cancer Institute (http://microarrays.nki.nl//download/files/operon_hs_060614.xls). A description of the annotation methodology used by The Netherlands Cancer Institute is provided on their Web site (http://microarrays.nki.nl/services/blastdata.html). We have only used those oligonucleotides that specifically respond in a BLAST search with a single hit on a gene. Expression values of multiple oligonucleotide probes targeting the same gene (identical gene symbol) were averaged, resulting in a total of 15,909 unique genes for further analysis. Subsequently, expression data obtained from Cy5- and Cy3-labeled samples of the same tumor were averaged (mean correlation 0.93 ± standard deviation 0.04). Microarray analyses were performed with the software package BRB Array Tools 3.6.0, developed by the Biometric Research Branch of the US National Cancer Institute (http://linus.nci.nih.gov/BRB-ArrayTools.html).

#### Survival prediction.

An overall survival profile was built using the supervised principal components method [23]. For each iteration of the complete cross-validation, 10% of the cases were omitted, and a subset of genes was selected that correlated with overall survival at a significance level of *p* < 0.001 for the remaining cases. The significance of each gene was measured based on a univariate Cox proportional hazards regression of survival time versus the log expression level. Next, PCA was performed to reduce the dimensionality of this selected subset of genes. Principal components (PCs), which are linear additions of weighted gene expression signals, were constructed in such a way that the first PC explained the largest amount of variance in our dataset and each subsequent PC explained the largest amount of the remaining variance while remaining uncorrelated with the previously constructed PC. Cox proportional hazards model was fitted to the data (with 10% of cases omitted) using the first five PCs as predictor variables, providing a regression coefficient (weight) for each principal component. The regression coefficients in combination with the first five PCs were used to calculate the predictive index for each sample. The 10% omitted test cases were classified as high or low risk based on whether their predictive index was above or below the median of the predictive indices for the 90% of cases in the training set. This entire procedure was repeated, leaving out a different 10% of cases until each case had been omitted exactly once and the cross-validated risk groups were determined for all cases. So the cross-validated risk group for each case was determined based on a predictor model that did not use that case in any way in its construction.

Kaplan-Meier survival curves were plotted for the predicted overall survival risk classes (high or low) giving a fair representation of the value of the expression profiles for predicting overall survival risk and the log-rank statistic was computed. To assess the significance of the log-rank statistic and the degree of overfitting, a phenotype permutation test based on 1,000 permutations was performed [24]. Survival data were randomly shuffled among the cases and the entire cross-validation process described above was repeated. For each random reshuffling, the process was repeated, new cross-validated Kaplan-Meier survival curves created, and the log-rank statistic for the random shuffling was computed, providing a null-distribution of the log-rank statistic. The tail area of this null distribution beyond the log-rank statistic obtained from the real data was the permutation significance level for testing the null hypothesis of no relation between the expression data and overall survival. A graphical representation of the survival prediction method is given in Figure S2.

The genes that are selected as univariately associated with survival will differ for each iteration of the cross-validation, because the entire predictor development process must be repeated from the beginning for each new cross-validated training set. The final gene set (profile) presented in the results was selected when the supervised principal components method was applied to the full dataset with no samples omitted.

Furthermore, to evaluate whether our profile provides more accurate predictions than that provided by standard clinicopathological covariates we performed a multivariate analysis using Cox proportional hazards regression.

#### Pathway and transcription factor analysis.

Functional gene set enrichment analysis was performed as described by Pavlidis et al. [25]. Predefined gene sets were analyzed to indicate which contained more genes correlated with overall survival than would be expected by chance. First a univariate Cox proportional hazards *p*-value was computed for all 15,909 unique genes. Then *p*-values of a subset of genes belonging to a functional set were summarized by the LS and KS summary statistics. For a set of *n* genes, the LS statistic is defined as the mean negative natural logarithm of single gene *p*-values. The KS statistic is defined as the maximum difference between i/*n* and *p*_i_, where *p*_i_ is the i^th^ smallest *p*-value. This is the Kolmogorov-Smirnov statistic for testing if the *p*-values are of uniform distribution. The statistical significance of a functional gene set containing *n* genes is evaluated by computing the empirical distribution of these summary statistics in random samples of *n* genes. A total of 167 functional gene sets reported in the Kyoto Encyclopedia of Genes and Genomes (KEGG) database and 111 reported in the Transcriptional Regulatory Element Database (TRED) were analyzed [26,27].

#### External validation.

To establish that our profile was associated with prognosis in an independent set of serous ovarian cancers, we used publicly available microarray data [4,9,28]. Available clinicopathologic characteristics of the 118 patients as reported previously are summarized in Table S1. Differences in microarray platforms (Operon V3 versus Affymetrix HU133A) meant that direct application of our prediction model on this independent dataset was not feasible [29]. The platforms differ, for example, in the reporter systems (short versus long oligonucleotides), labeling techniques, and hybridization protocols. Furthermore, many probes from the two platforms assigned to the same gene symbol are in fact detecting different splicing variants. Therefore, the genes composing our profile, but not the prediction rule, were generalizable to the independent dataset using a previously described methodology [30]. Probe sets from the Affymetrix HU133A microarray related to the genes in our profile were selected. Subsequently, we applied the survival prediction method, with cross-validation and permutation testing as described above, to assess the performance of a classifier for the independent dataset based only on these selected probes; however, this time a significance threshold of 0.9999 was used to ensure that all selected probes representing genes from our survival profile were used in each iteration of the cross-validation. Furthermore, functional class scoring analysis for pathways and transcription factors as described above using all available probes was also performed on this independent dataset to compare results [25].

#### Oncogenic pathway activation analysis.

Recently, Bild et al. experimentally generated expression signatures that reflect the activation of various oncogenic signaling pathways, and provided software to assess the activation status in individual expression profiles [28]. We used these publicly available expression profiles and software to assess the activation probability of the c-Myc, H-Ras, c-Src, E2F3, and β-catenin pathway in our 157 ovarian tumor samples. For each pathway we divided the tumor samples into a group with an activation probability *p* < 0.5 and a group with activation probability *p* > 0.5. Next, Kaplan-Meier survival curves were plotted for both groups and the log-rank statistic computed. A significant log-rank statistic would indicate an association between the activation status of the oncogenic pathway and overall survival. Furthermore, to assess the combined effect of the five oncogenic pathways on overall survival, we applied average linkage hierarchical clustering according to the uncentered correlation measure on the activation probabilities. Kaplan-Meier survival curves were plotted for the top two clusters and the log-rank statistic was computed.

## Results

### Patient Characteristics

Clinicopathologic characteristics of the 157 platinum-treated patients with serous ovarian cancer are summarized in Table 1. For the whole group the median overall survival time was 21 mo (range 1–234 mo), and the 5-y overall survival rate was 27%.

**Table 1:**
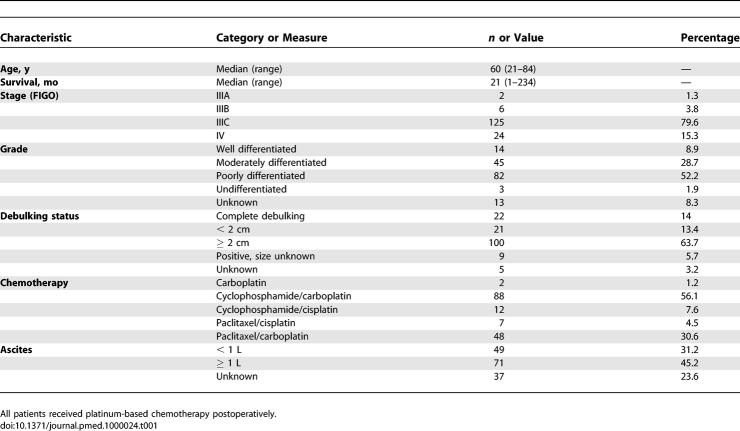
Patient Characteristics (*n* = 157)

### Gene Expression Profile Associated with Overall Survival

**Figure 1:**
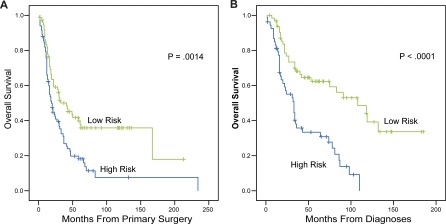
Kaplan-Meier Survival Curves for the Patients Predicted to Have Above or Below Median Risk of Death Due to Ovarian Cancer (A) Present study, median survival of 19 mo versus 41 mo (*p* = 0.0014, log-rank), permutation *p*-value = 0.015. (B) Dressman et al. [9], median survival of 33 mo versus 108 mo (*p* < 0.0001, log-rank), permutation *p*-value = 0.007.

Eighty-six genes were found to correlate with overall survival by univariately fitting Cox proportional models at an alpha of 0.001. In Table 2 each gene is listed with its *p*-value, cross-validation support, hazard ratio (HR), and description. Univariate *p*-values, HR, and false discovery rates (FDRs) for all 15,909 genes are given in Table S2. The FDR associated with a row of the table is an estimate of the proportion of the genes with univariate *p*-values less than or equal to the one in that row that represent false positives [31]. Figure 1A shows the Kaplan-Meier survival curves for the cross-validated risk groups predicted to have above (*n* = 83) or below (*n* = 74) median risk of death due to ovarian cancer. Table S3 contains the necessary weights and final prediction rule to calculate the predictive index for a sample based on the expression signals of the identified 86 genes. As mentioned in the methods section the cross-validated risk group for each case was determined based on a predictor model that did not use that case in any way in its construction. The low-risk group had a median survival of 41 mo, whereas the high-risk group had a median survival of 19 mo (*p* = 0.0014, log-rank). The permutation test based on 1,000 permutations resulted in a *p*-value of 0.0015, indicating that the chance that such a log-rank static is based on overfitting is small. Table 3 shows the distribution of several prognostic factors as a function of risk assignment based on our 86-gene overall survival profile. Age, stage, grade, debulking status, and chemotherapy regimens showed no difference in distribution between the predicted low- and high-risk groups. In addition to our overall survival profile, only the amount of residual tumor after primary surgery (*p* = 0.0003; HR = 2.34) showed a prognostic value for overall survival in univariate analysis. In multivariate analysis our overall survival profile (*p* = 0.008; HR = 1.94; 95% confidence interval [CI] 1.19–3.16) and debulking status (*p* = 0.006; HR = 2.44; 95% CI 1.30–4.60) both maintained independent prognostic significance (Table 4).

**Table 2:**
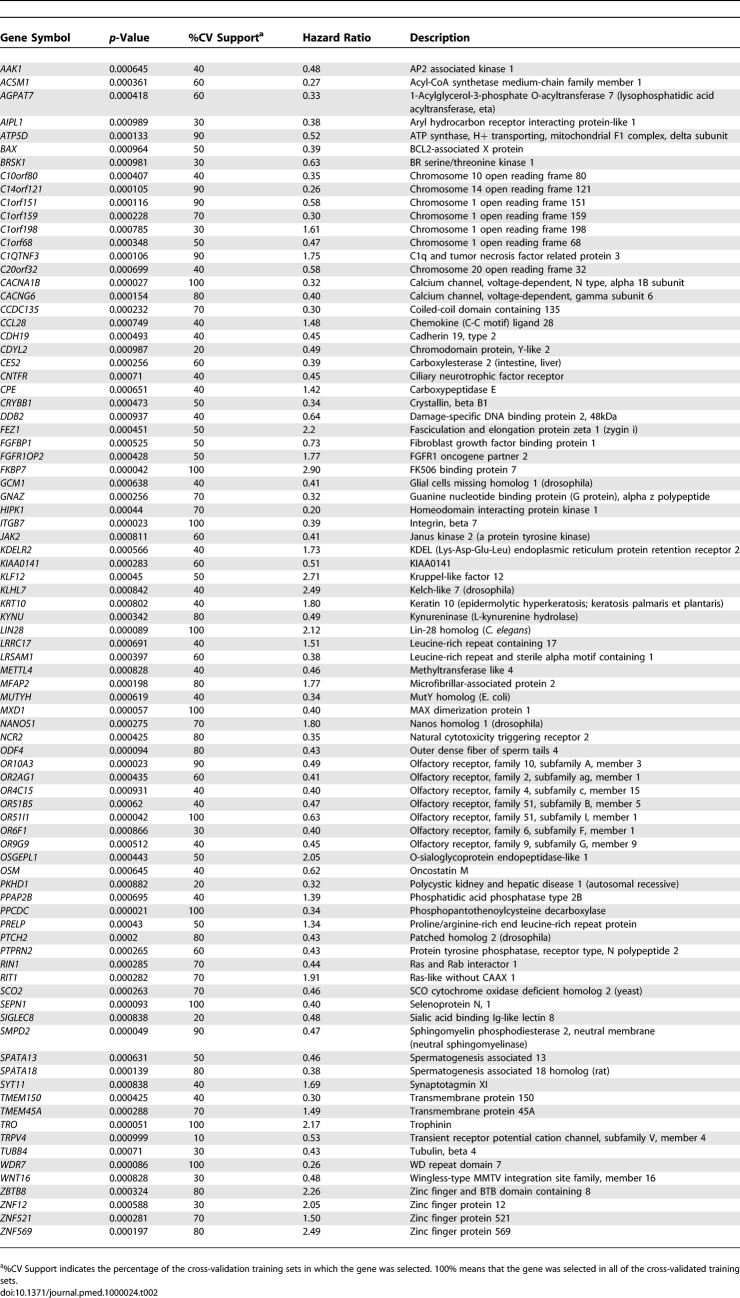
Genes Composing the Overall Survival Profile

**Table 3:**
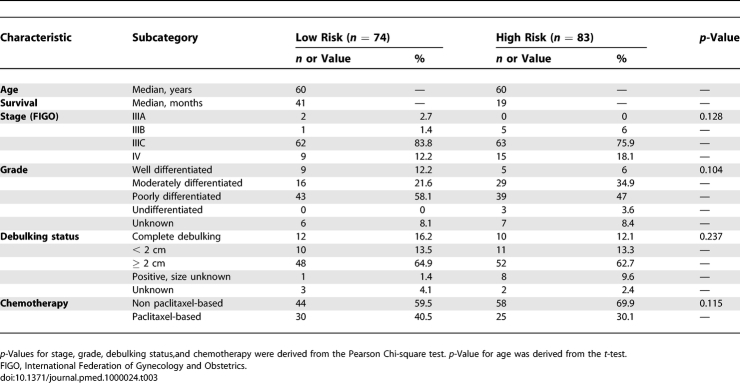
Association between the Overall Survival Profile and Clinicopathologic Characteristics

**Table 4:**
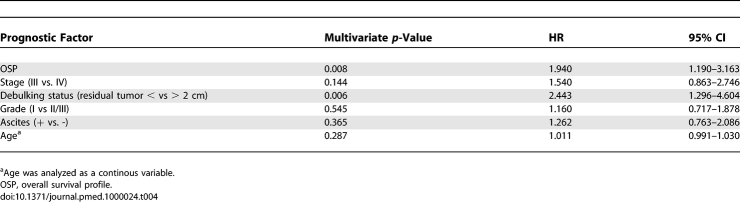
Prognostic Value of the Overall Survival Profile Adjusted for Debulking Status, Stage, Grade, Age, and Ascites by Cox Proportional Hazards Regression

### QRT-PCR Validation of the Overall Survival Profile

**Figure 2:**
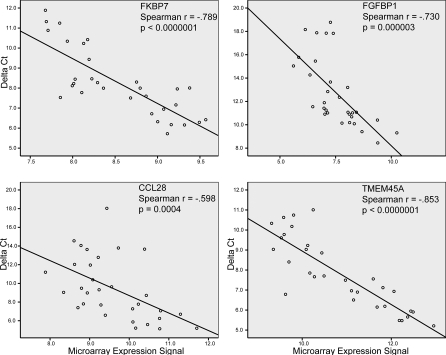
Scatter Plots Showing the Microarray Expression Signal Versus the ΔCt Obtained by qRT-PCR for Four Individual Genes Genes are *FGFBP1*, *FKBP7*, *TMEM45A*, and *CCL28* from our overall survival profile. ΔCt of the gene is obtained by subtracting the mean Ct value of *GAPDH* from the mean Ct value of the gene. Both axes are on log_2_ scale.

To validate the overall survival profile, we performed qRT-PCR for 31 total RNA samples that had been included in the microarray analysis. Four genes, i.e., *FGFBP1*, *FKBP7*, *TMEM45A*, and *CCL28*, that differ in percentage cross-validation support were arbitrarily selected from the 86-gene overall survival profile. Relative expression levels for each gene were correlated with the corresponding microarray signal intensities. A strong correlation between qRT-PCR and microarray signal intensities was observed for all four genes (Figure 2).

### Independent Validation of the Overall Survival Profile

Because of the different microarray platforms used, our predictor model could not be directly tested on the independent serous ovarian cancer dataset. Instead we identified 97 probes on the HU133A platform, targeting 57 out of the 86 unique genes from our overall survival profile. These probes were used to build a survival profile as described above. Figure 1B shows the Kaplan-Meier survival curves for the patients from the independent dataset predicted to have above or below median risk of death due to ovarian cancer. This low-risk group had a median survival of 108 mo, whereas the high-risk group had a median survival of 33 mo (*p* < 0.0001, log-rank). The permutation *p*-value of the log-rank test statistic between the two risk groups, based on 1,000 permutations, was *p* = 0.007.

### Pathways and Transcription Factors Associated with Overall Survival

As input for the survival gene set analysis, significance levels based on univariate Cox proportional hazards regression of survival time versus the log expression level for all 15,909 genes were used. KEGG pathways and transcription factors associated with overall survival in our dataset and that of Dressman et al. [9] are shown in Tables 5 and 6, respectively. In our dataset 17/167 pathways and 13/111 transcription factors were associated with overall survival, of which 16 and 12, respectively, were confirmed in the independent dataset from Dressman et al. [9]. Table S4 shows univariate *p*-values and hazard ratios for genes that form part of the 17 pathways associated with overall survival in our dataset.

**Table 5:**
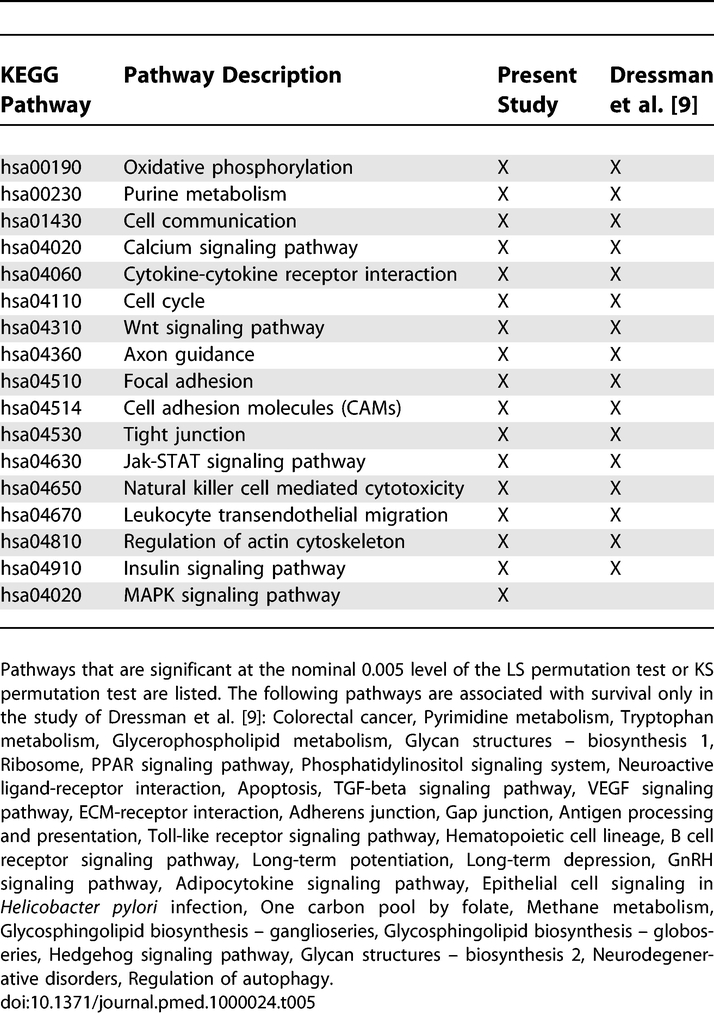
KEGG Pathways with More Genes Correlated with Overall Survival Than Expected by Chance as Identified in the Present Study and in the Dressman et al. [9] Dataset

**Table 6:**
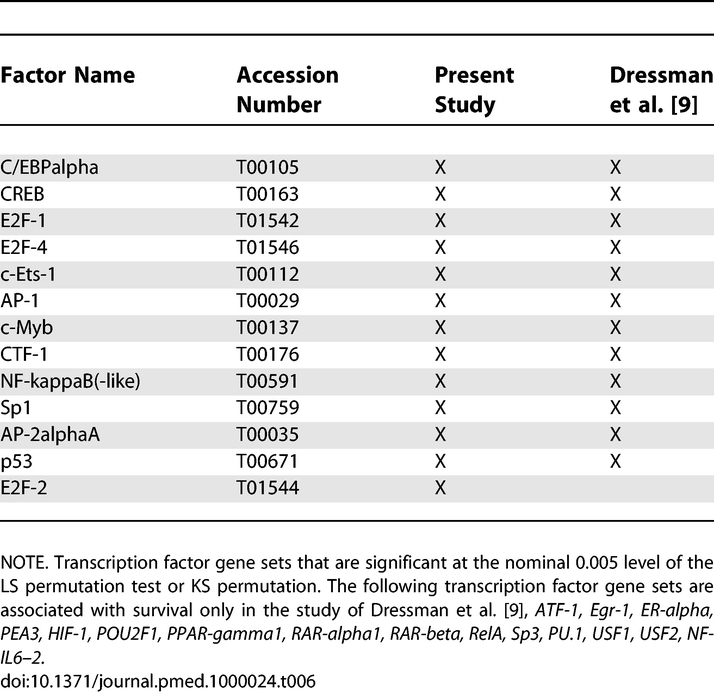
Transcription Factor Gene Sets with More Genes Correlated with Overall Survival Than Expected by Chance as Identified in the Present Study and in the Dressman et al. [9] Dataset

### Oncogenic Pathway Activation

**Figure 3:**
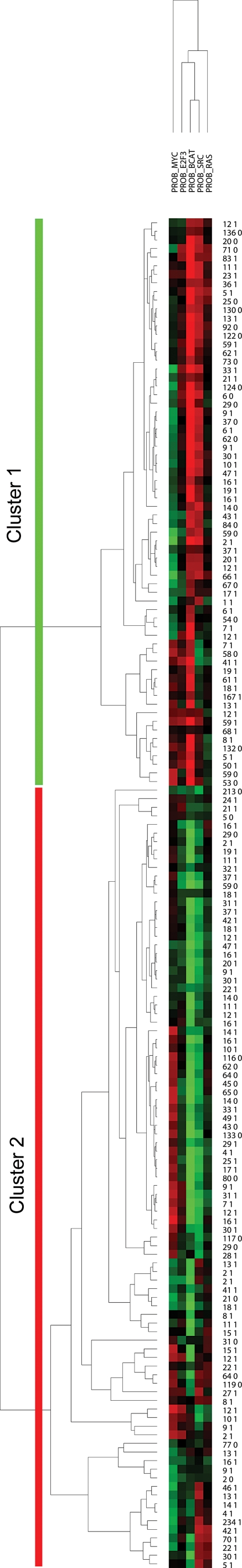
Heatmap Showing Predicted Probabilities of Pathway Activation for the Five Oncogenic Pathways Pathways are c-Myc, H-Ras, c-Src, E2F3, and β-catenin in our 157 ovarian tumor samples. Red indicates a high probability of activation and green indicates a low probability of activation. The probabilities of pathway activation were clustered according to the uncentered correlation measure. On the right of the figure the follow-up in months is depicted followed by the censoring status for each of the 157 samples.

Figure 3 shows a heatmap of the predicted activation status for the five oncogenic pathways in our 157 ovarian tumor samples. Red indicates a high probability of activation and green indicates a low probability of activation. None of the five oncogenic pathways showed a significant log-rank statistic for the Kaplan-Meier survival curves between the group of ovarian tumor samples with an activation probability *p* < 0.5 and the group with an activation probability *p* > 0.5. The five Kaplan-Meier curves and the associated log-rank *p*-values are shown in Figure S3. After clustering the samples on their activation probabilities for the five oncogenic pathways combined, the top two clusters showed no significant difference between their Kaplan-Meier survival curves (Figure S4).

## Discussion

In this study on tumors of a large series of well-documented advanced-stage serous ovarian cancer patients we identified a gene expression profile that reflects patients' overall survival. Our overall survival profile maintained independent prognostic significance in multivariate analysis. Moreover, expression of the genes that composed our overall survival profile also held their prognostic value in an independent dataset processed at a different institution using a different microarray platform. Finally, in addition to individual genes, we were also able to reproducibly identify and validate KEGG pathways and TRED transcription factors associated with overall survival.

Limited overlap with respect to individual genes, as observed in comparable studies in other tumor types, was also found between our overall survival profile and those reported in three previous ovarian cancer microarray studies [4,7,32].

Nonreproducibility of prognostic profiles between different microarray studies in the same tumor type can be attributed to a variety of methodological issues [33,34]. Our study specifically pays attention to methodological principles such as randomization and replication. Thus, confounding effects are avoided and unbiased estimation of differential expression levels is provided. The three studies mentioned above did not include a replication of the experiment [4,7,32]. In contrast to these studies, by using supervised PCA to build prognostic profiles, we could consider survival a continuous parameter, and patients were thus not forced into subgroups that might not be biologically meaningful. Categorizing patients a priori into a “low risk” and “high risk” subgroup based on survival times might cause any future predictions based on this model to be suspect when underlying, unidentified, biologically different subgroups have considerable overlap in survival times.

Our overall survival profile contains several interesting genes (Table 2) that offer potential insight into mechanisms associated with tumor behavior. The overall validity of our approach is confirmed by the observation that for some of the genes in our profile, earlier studies that utilized non–array-based methodologies already indicated their relevance in ovarian cancer. For example, high expression of the proapoptotic *BAX* gene was associated with improved prognosis in our study. Previously, a similar relation between *BAX* expression and response to chemotherapy and overall survival in ovarian cancer was reported by others [8,35–37]. Likewise, in our study, high expression of Ras inhibitor 1 (*RIN1*) and low expression of Ras-like without CAAX1 (*RIT1*) were associated with better overall survival, which is in agreement with previous work by others showing that activated Ras contributes to the maintenance and growth of ovarian carcinomas [38]. In addition to these already known relevant genes in ovarian cancer our study reveals also multiple new genes with a possible impact on tumor behavior in ovarian cancer. For example *OSM*, *JAK2*, and *CNTFR* are components of the Jak/STAT signaling pathway, which can stimulate cell proliferation, differentiation, cell migration, and apoptosis [39]. *OSM* has individually been identified as a potent suppressor of tumor cell proliferation and inducer of differentiation in multiple tissues [40]. *FGFBP1* and *FGFR1* interact with fibroblast growth factors *FGF1* and *FGF2*, and it has been suggested that the fibroblast growth factors could serve as the angiogenic switch in human cancer [41–43]. Apart from having prognostic impact our analysis also sheds light on previous unknown possible “druggable” targets in ovarian cancer; *FKBP7,* with the highest hazard ratio for individual genes in our study, appears to be especially of interest because FKBPs can be targeted with mTOR inhibitors [44].

Although individual genes such as those described above may prove to be relevant for tumor behavior, it is often not known in ovarian and other cancers whether large fold-changes in individual genes will have more biological relevance than will smaller but coordinated fold-changes in a set of genes along a single pathway. Analysis of our microarray data by integration of genes into functional gene sets according to well-known biological pathways and transcription factors enabled us to consider all available genomic information rather than only genes passing a certain significance threshold, thus providing extra clues to which signaling pathways and transcription factors contribute to the clinical outcome of ovarian cancer. Bonome et al. [32] recently also identified possible signaling events (pathways). However, by using classical over-representation analysis of functional gene sets within only a small subset of genes (57 probes) associated with overall survival, the authors disregarded most of the genomic information available [32].

Our pathway analysis, which to our knowledge has not been applied to ovarian cancer array datasets previously, revealed 17 pathways to be related to survival, of which 16 were validated in the independent dataset. As previously mentioned, there is usually only limited overlap with respect to individual genes between the prognostic profiles previously published in (ovarian) cancer [45]. In contrast, integrating genes in functional gene sets according to pathways and transcription factors in this study resulted in considerable overlap between our dataset and the independent dataset with respect to prognostic impact. Our results therefore indicate that assessment of prognostic profiles is more robust by determination of coordinated expression of several signaling pathways than of expression of individual genes.

Thus in the present study, cell cycle, Wnt, Jak-STAT, and MAPK pathways, playing a role in apoptosis, proliferation, differentiation, and/or cell cycle, were identified as having a role in ovarian cancer; consistent with these results, aberrant signaling of each of these pathways has been proposed as contributing to ovarian carcinogenesis [46–50], again indicating the strength of our approach. Novel therapeutic options are currently being explored that act on several signaling pathways that we found to be associated with overall survival in ovarian cancer. For example, Basica et al. recently described activation of Wnt signaling in ovarian and breast cancer cell lines and showed that Wnt antagonists dramatically altered the biological behavior of these cells [51]. A blocking antibody against FZD10, a cell membrane receptor for Wnt, was shown to have strong antitumor effect in xenografted tumors overexpressing FZD10 [52,53]. As in our study, a dysregulated Wnt pathway and, more specifically, overexpression of FZD10 and FZD7 (another member of the Wnt cell-surface receptor family) were related to worse prognosis, so this FZD10 blocking antibody may have clinical potential by inhibiting the autocrine Wnt signalling pathway in ovarian cancer.

Our transcription factor analysis also implicated several transcription factors (Table 5) in ovarian cancer. With different methodologies A2P-2alpha and c-Ets-1 have previously been associated with poor prognosis in ovarian cancer [54–56]. Similarly, E2F transcription factors, members of the C/EBP family, and CREB were demonstrated to play an important role in ovarian carcinogenesis [57–60]. With the exception of p53, most transcription factors are presently difficult to target. Our transcription factor analysis demonstrated a positive prognostic impact for p53 as an “activated” transcription factor. In tumor cell lines activation of wild-type p53 can be induced by the small molecule nutlin-3, which antagonizes the function of the natural inhibitor of wild-type p53 MDM2, resulting in enhanced apoptosis or cell cycle arrest [61,62]. In combination, these observations tempt one to speculate on a possible role for nutlin-3 in overcoming resistance to chemotherapy, especially in those ovarian cancers with a less activated p53 [61,63].

A limitation of functional gene set enrichment analysis as performed in our study, however, is its inability to assess the activation status of identified pathways in an individual tumor sample. This gap is potentially filled by the strategy described by Dressman et al. [9] and Bild et al. [28] (see also Materials and Methods: Oncogenic pathway activation analysis). In this study we assessed the activation status of five oncogenic signaling pathways in 157 ovarian tumor samples based on the activation observed in a mammalian cell line following tranfection of adenovirus expressing human c-Myc, activated H-Ras, human c-Src, human E2F3, or β-catenin. Bild et al. showed in a series of 153 samples that activated E2F3, β-catenin, and c-Src pathways were associated with poor overall survival in ovarian cancer [28]. None of these five oncogenic pathways showed a significant association (as individual pathways and/or in combination) with overall survival in our dataset (see Figures S3 and S4). Our results therefore indicate that analysis of pathway activation status until now is not very robust and cannot be easily exchanged between different studies, as was recently also described by others [64].

In this study the prognostic impact of 57 genes from our profile was validated within the independent dataset from Dressman et al. [9], providing stronger evidence than before that these genes are important in the biological behavior of ovarian tumors. Platform differences did not allow one-to-one exact validation of the underlying predictive algorithm. Therefore, the clinical usefulness of our overall survival predictor needs further validation. With regard to the reproducibility issues between different microarray platforms, it seems likely that in the future an array-based prognostic tool will be based on a single platform. So, our study might be considered a phase II study in which we constructed a genetic profile associated with overall survival within the whole group of 157 patients according to statistical principles such as cross-validation and permutation testing [65]. As described above, such a study provides valuable clues about the mechanisms underlying tumor behavior and clues with respect to potential drug development targets (genes, pathways, etc). To further evaluate the prognostic impact of our profile for individual patients, prospective studies need to be performed. Another important issue that needs to be addressed in such studies is the impact of intratumor heterogeneity on reproducibility. Using a combination of microsatellite analysis and SNP analysis Khalique et al. recently showed that intratumor heterogeneity is a common feature within epithelial ovarian cancer [66].

In conclusion, our study provides new, validated insights into molecular changes in genes, pathways, and transcription factors that are relevant for ovarian cancer behavior and that should therefore be exploited in the search for new treatment strategies towards patient-tailored therapy. In the future, pathway activation analysis in individual tumors may guide the choice of targeting drugs in ovarian cancer patients [67,68], but its methodology needs to become more robust before clinical relevance can be envisioned.

## Supporting Information

Figure S1 Diagram Showing the Randomization Design (497 KB TIF)

Figure S2 A Graphical Representation of the Survival Prediction Method (2.6 MB TIF)

Figure S3 Kaplan-Meier Survival Curves for the Five Oncogenic Pathways between the Group of Ovarian Tumor Samples with an Activation Probability *p* < 0.5 and the Group with an Activation Probability *p* > 0.5 (1.5 MB TIF)

Figure S4 Kaplan-Meier Survival Curves of the Top Two Clusters after Clustering the Samples on their Activation Probabilities for the Five Oncogenic Pathways Combined (1 MB TIF)

Table S1 Available Clinicopathologic Characteristics of the 118 Patients as Reported by Dressman et al. [9] (35 KB DOC)

Table S2 Overall Survival-Related Univariate *p*-Values, HR, and FDR for all 15,909 Genes (1.5 MB XLS)

Table S3 Weights and Final Prediction Rule to Calculate the Predictive Index for a Sample Based on the Expression Signals of the Identified 86 Genes (41 KB XLS)

Table S4 Univariate *p*-Values and Hazard Ratios for Genes that Form Part of the 17 Pathways Associated with Overall Survival in Our Dataset (266 KB XLS)

## Abbreviations

CI: confidence interval
Ct: comparative threshold
FDR: false discovery rate
HR: hazard ratio
KEGG: Kyoto Encyclopedia of Genes and Genomes
PC: principal component
PCA: principal components analysis
qRT-PCR: quantitative real-time-PCR
