# Supplementary material for: Survival-Related Profile, Pathways, and Transcription Factors in Ovarian Cancer
Source: PLoS Med. 2009 Feb 3;6(2):e1000024. doi: 10.1371/journal.pmed.1000024 (PMC2634794; doi:10.1371/journal.pmed.1000024)
Supplement: Supplementary file 1 [file pmed.1000024.st001.doc]

**Table S1**. Patient characteristics (N = 118).

| Characteristic |  | N |  | % |
| --- | --- | --- | --- | --- |
| Age (tN = 52) | Median (years) |  | 60 |  |
|  | Range |  | 33-79 |  |
| Stage (tN = 117) | III | 98 |  | 84.5 |
|  | IV | 18 |  | 15.5 |
| Grade (tN = 115) | 1 | 4 |  | 3.5 |
|  | 2 | 55 |  | 47.8 |
|  | 3 | 55 |  | 47.8 |
|  | Undifferentiated | 1 |  | 0.9 |
| Debulking status  (tN = 118) | Optimal, < 1 cm | 63 |  | 53.4 |
|  | Suboptimal, >= 1 cm | 55 |  | 46.6 |
| Survival  (tN = 118) | Median (months) |  | 34 |  |
|  | Range |  | 1-185 |  |

Abbreviations: tN, total number of patients of which clinicopathologic data was available.
